# Influence of Bentall Procedure on Left Ventricular Function

**DOI:** 10.21470/1678-9741-2019-0147

**Published:** 2020

**Authors:** Serkan Burç Deşer, Mustafa Kemal Demirag, Semih Murat Yucel, Ufuk Yildirim, Murat Muzaffer Güçlü, Merve Polat, Fersat Kolbakir, Hasan Tahsin Keceligil

**Affiliations:** 1 Department of Cardiovascular Surgery, School of Medicine, Ondokuz Mayis University, Samsun, Turkey.; 2 Department of Cardiology, School of Medicine, Ondokuz Mayis University, Samsun, Turkey.

**Keywords:** Ventricular Function, Left, Survival Rate, Atrial Fibrilation, Aortic Valce, Echocardiography, Diastole, Aorta

## Abstract

**Objective:**

To evaluate the influence of Bentall procedure on left ventricular function and condition on long-term follow-up.

**Methods:**

Seventy-three consecutive patients who underwent an aortic root and ascending aorta replacement with composite valve button Bentall or flanged Bentall technique, from January 2007 to November 2018, were included in this retrospective study.

**Results:**

Postoperative left ventricular ejection fraction significantly increased (52.14±11.38 *vs*. 56.79±11.36; *P*=0.041), left ventricular end-systolic diameter significantly reduced (38.25±9.31 mm *vs*. 34.17±9.15 mm; *P*=0.027), left ventricular end-diastolic diameter significantly reduced (56.42±9.72 mm *vs*. 51.58±9.03 mm; *P*=0.01), and left atrial diameter significantly reduced (45.33±12.77 mm *vs*. 39.25±12.41 mm; *P*=0.01), compared to preoperative values. Our long-term survival results are comparable with previous studies in which survival rates in 5 years and 10 years were 83.5% and 69.8%, respectively. In comparing patients according to their New York Heart Association (NYHA) functional class, it was shown that their postoperative functional capacity was improved during the follow-up period (2.1±0.56 *vs*. 1.2±0.42; *P*=0.001).

**Conclusion:**

The Bentall procedure significantly improved the left ventricular systolic function and condition and decreased the left ventricular end-systolic and end-diastolic diameters and the left atrial diameter on long-term follow-up, based on the transthoracic echocardiography. Bentall procedure can be performed with acceptable mortality and morbidity rates on long-term follow-up.

**Table:** 

Abbreviations, acronyms & symbols	
AVR	= Aortic valve replacement
BMI	= Body mass index
ICU	= Intensive care unit
INR	= International normalized ratio
LV	= Left ventricular
LVEF	= Left ventricular ejection fraction
NYHA	= New York Heart Association
SD	= Standard deviation
SPSS	= Statistical Package for the Social Sciences

## INTRODUCTION

The yearly incidence of mediastinal and thoracic aortic aneurysm is estimated to be about 4.5/100.000 in total population and 60% of them are related to the aortic root and ascending aortic aneurysm^[1]^. Early surgery is recommended due to dissection or rupture risk^[2]^. Aortic root and valve replacement with a composite graft with a mechanical valve and coronary artery reimplantation were first described in 1968 by Bentall and de Bono^[3]^. Since then, Bentall procedure has been the treatment of choice for aortic root with or without ascending aorta aneurysms, which improves clinics and hemodynamics of patients^[3]^. Button Bentall technique has been evolved and modified by Kouchoukos, as in 1981^[4]^. David reimplantation^[5]^ and Sarsam and Yacoub remodeling techniques have been described as alternative valve sparing techniques^[6]^. Several modifications, such as button or flanged Bentall, were evolved to reduce bleeding, reduce coronary button tension, avoid kinking of coronary arteries, and reduce the time of surgery^[3,7]^. Coronary button separation, bleeding, and false aneurysm are the potential complications, which can be reduced with Cabrol procedure^[8]^. Mechanical valves are recommended for patients younger than 50 years old and bioprosthetic valves are recommended for patients older than 70 years old, which reduces the risk of thromboembolism, hemorrhage, and endocarditis. Besides that, the type of valve should be chosen regarding the expected survival, comorbidities, and risk of surgery^[9]^. Urgent Bentall procedure should be performed for the diagnosis of acute type A aortic dissection, prosthetic graft infection, aortic valve endocarditis, and aortic abscess, while elective surgery should be performed for annuloaortic ectasia, aortic valve regurgitation, collagen tissue defects, such as Marfan syndrome, Loeys Dietz syndrome, Ehler Danlos syndrome, chronic type A aortic dissection, and post-stenotic dilatation^[10,11]^. Data regarding left ventricular (LV) function and condition after the Bentall procedure on long-term follow-up are scarce. In this retrospective study, we aimed to evaluate and analyze the left ventricular ejection fraction (LVEF) and condition after elective Bentall procedure on long-term follow-up.

## METHODS

### Study Population

**Fig. 1:**
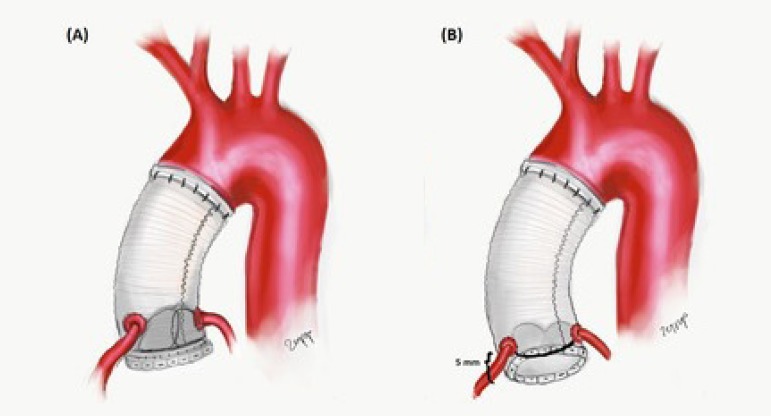
Depiction of button Bentall (A) and flanged Bentall (B) procedures.

Seventy-three consecutive patients who underwent aortic root with ascending aorta replacement with composite valve button Bentall or flanged Bentall technique, from January 2007 to November 2018, were included in this retrospective study (Figure 1). Perioperative risk factors, postoperative outcomes (stroke, bleeding, acute renal failure, etc.), 30-day operative mortality and mortality rates, mid-term and long-term survival, comorbidities (diabetes mellitus, hypertension, chronic obstructive pulmonary disease, coronary artery disease, Marfan syndrome, history of cardiac surgery, functional capacity, hyperlipidemia, chronic renal failure, etc.), need for concomitant procedures (*i.e*., coronary artery bypass grafting, mitral valve replacement, and hemiarch replacement), need for hypothermic circulatory arrest, overall in-hospital stay, intensive care unit (ICU) stay, and etiology were analyzed. All data including the demographic and clinical features of the patients were retrieved from the hospital’s database. Of the 73 patients, long-term follow-up rate was 69.8% (51 patients). Physical examination, computed tomography scans, and transthoracic echocardiography were performed in all patients under follow-up. Patients with acute DeBakey type A aortic dissection and patients who underwent redo Bentall procedure were excluded. The presence of heart failure was defined in terms of the New York Heart Association (NYHA) functional classification. Clinical follow-up is performed every month with international normalized ratio (INR), and every six months with transthoracic echocardiography and computed tomography angiography. Table 1 summarizes the patients’ preoperative variables and operative data.

**Table 1:** Patients’ preoperative variables and operative data.

Preoperative variables and operative data	n (%)	Mean±SD
Age (years)		57.82±15.28
Sex, male	36 (70.5)	
Weight (kg)		74±9.8
BMI		24.6±4.6
Hypertension	39 (76.4)	
Hyperlipidemia	7 (13.7)	
Diabetes mellitus	5 (9.8)	
Smoking	10 (19.6)	
Chronic obstructive pulmonary disease	6 (11.7)	
Coronary artery disease	7 (13.7)	
Chronic renal disease	2 (3.9)	
Bicuspid aortic valve	5 (9.8)	
Marfan syndrome	5 (9.8)	
Ejection fraction < 50 %	12 (23.5)	
Mechanical valve	46 (90.2)	
Bioprosthetic valve	5 (9.8)	
Composite valve size (mm)	23.07±1.05	
Concomitant cardiac procedures		
Mitral valve replacement	3 (5.8)	
Coronary artery bypass	7 (13.7)	
Hemiarch replacement	5 (9.8)	

BMI=body mass index; SD=standard deviation

### Surgical Method

Flanged Bentall or button Bentall modification was performed in all patients (Figure 1). In the Bentall and de Bono button technique, which was first described in 1968^[3]^, after the ascending aorta and aortic valve were resected, a composite graft was created with a mechanical aortic valve and a tubular graft was sutured. Then the composite graft was sutured to the aortic annulus with continuous polypropylene sutures. In the flanged Bentall procedure, which was first reported by Yakut in 2001^[12]^, after approximately 5 mm of the end of the tubular graft is evaginated, the mechanical valve is sutured to the graft with continuous polypropylene sutures. Then the conduit was sutured to the aortic annulus from the everted part (flanged) of the graft with continuous polypropylene sutures (Figure 1). Distal anastomosis was performed with either closed or open technique, using hypothermic circulatory arrest alone or with selective antegrade or retrograde cerebral perfusion, according to the need. The partial cardiopulmonary bypass was instituted with ascending aortic cannulation or right axillary artery cannulation and two-stage venous cannulation via median sternotomy. Femoral cannulation was instituted in redo cases. Routine antegrade cardioplegia was used for the constituted diastolic arrest, then retrograde cardioplegia was used routinely to maintain the diastolic arrest and topical cooling was applied for myocardial protection. Deep hypothermic circulatory arrest (18ºC) was achieved with antegrade cerebral perfusion in selective cases and open clamp technique was performed for distal anastomosis. Both mechanical and bioprosthetic valves were used. The composite valve graft was created with Dacron tube graft and St. Jude valves (St. Jude Medical, Inc., St. Paul, Minnesota, USA), Carbomedics valve (Carbomedics, Austin, Texas, USA), or Sorin valves (Sorin, Milano, Italy), with a continuous 4-0 poly-fluoride vinyl suture. Of the 51 patients, 21-mm aortic valves with composite graft were implanted in six patients, 23-mm aortic valves with composite graft were implanted in 37 patients, and 25-mm aortic valves with composite graft were implanted in eight patients. The mean sinotubular junction was 50.1±2.7 mm. Proximal anastomosis was performed with pledgeted 3-0 polyester sutures, while distal anastomosis (4-0) and coronary button anastomoses (6-0) were performed with continuous poly-fluoride vinyl sutures. Postoperative low-molecular-weight heparin and warfarin sodium were administered to all patients. Anticoagulation was achieved with an INR between 2,5 and 3,5.

### Follow-Up

Transthoracic echocardiographic examinations were performed with Vivid® 7 (GE Healthcare, Waukesha, Wisconsin, USA) before surgery and two weeks, one, three, and six months after surgery, and then performed annually. For this study, the remaining 51 patients were called, and transthoracic echocardiography was performed by the same cardiologist with the same device Vivid® 7 (GE Healthcare, Waukesha, Wisconsin, USA). LV end-systolic and end-diastolic diameters, LVEF (using a modified Simpson biplane formula), interventricular septum, pulmonary artery pressure, right ventricular diameter, left atrial diameter, and posterior wall diameter were calculated. Patients’ functional statuses were defined on admission and after a mean period of 31±27.3 months (range 3-96 months) according to the NYHA functional classification. Overall survival was analyzed.

The study protocol was approved by the local ethics committee (OMU KAEK 2019/75). The study was carried out in accordance with the Helsinki Declaration principles.

### Statistical Analysis

The Statistical Package for the Social Sciences software for Windows (SPSS Inc, Chicago, Illinois, USA), version 21, was used to compare the data. The Kolmogorov-Smirnov test was used to analyze normally distributed continuous variables. Categorical variables were presented in percentages and frequencies. The categorical data were tested with the Chi-square test or Fisher’s exact test. Continuous variables were presented in mean ± standard deviation (SD). The continuous variables were compared using the *t*-test and the Mann-Whitney U test. Kaplan Meier test was performed for cumulative survival. A *P*-value of <0.05 was considered statistically significant.

## RESULTS

### Sample Sizes and Demographic Features

**Fig. 2:**
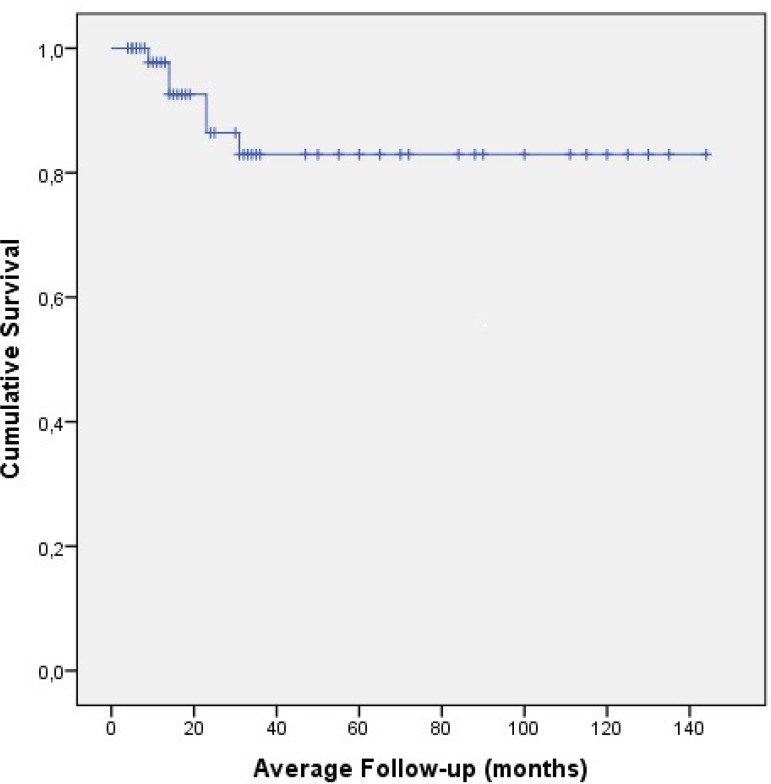
Cumulative survival rate of patients.

A total of 51 patients (36 males, 15 females; mean age 57.82±15.28 years [range 14-90 years]) were followed up out of 73 patients who underwent aortic root with ascending aorta replacement with composite valve button Bentall or flanged Bentall technique; transthoracic echocardiography was performed. Of the 73 patients, the mean length of follow-up was 31±27,3 months (range 3-96 months) (Figure 2).

In the 51 patients, comparing to preoperative values, postoperative LVEF has significantly increased (52.14±11.38 *vs*. 56.79±11.36; *P*=0.041), LV end-systolic diameter has significantly reduced (38.25±9.31 mm *vs*. 34.17±9.15 mm; *P*=0.027), LV end-diastolic diameter has significantly reduced (56.42±9.72 mm *vs*. 51.58±9.03 mm; *P*=0.01), and left atrial diameter has significantly reduced (45.33±12.77 mm *vs*. 39.25±12.41 mm; *P*=0.01), while postoperative right ventricular diameter has reduced (29.25±4.57 mm *vs*. 29.08±5.60 mm; *P*=0.086) without statistically significance (Table 2).

**Table 2:** Comparison between preoperative and postoperative transthoracic echocardiographic parameters.

	Preoperative Mean±SD	Postoperative Mean±SD	*P*-value
Ejection fraction	52.14±11.38	56.79±11.36	0.041
Left ventricular end-systolic diameter (mm)	38.25±9.31	34.17±9.15	0.027
Left ventricular end-diastolic diameter (mm)	56.42±9.72	51.58±9.03	0.01
Interventricular septum (mm)	14.33±3.96	13.42±1.73	0.13
Posterior wall diameter (mm)	12.58±3.02	12.42±1.83	0.75
Left atrial diameter (mm)	45.33±12.77	39.25±12.41	0.01
Right ventricular diameter (mm)	29.25±4.57	29.08±5.60	0.086
Pulmonary artery pressure	29.08±6.07	28.38±4.8	0.51

SD=standard deviation

On contrast, postoperative pulmonary artery pressure (29.08±6.07 *vs*. 28.38±4.8; *P*=0.51), posterior wall diameter (12.58±3.02 mm *vs*. 12.42±1.83 mm; *P*=0.75), and interventricular septum (14.33±3.96 mm *vs*. 13.42±1.73 mm; *P*=0.13) have not significantly changed after index surgery.

Of the 73 patients, the overall early mortality (six patients), late mortality (six patients), and major stroke (two patients) rates were 8.3%, 8.3%, and 2.8%, respectively, and eight patients were out of follow-up.

Of the remaining 51 patients, five patients (9.8%) underwent hemiarch replacement, postoperative bleeding was observed in five patients (9.8%), and no thromboembolic complication was seen. All patients had aortic valve insufficiency, the annuloaortic ectasia with ascending aorta aneurysm was found to be the most common etiology in our study (46 patients, 90.2%) and the remaining five patients (9.8%) had collagen tissue defects (Marfan syndrome). When we compared patients according to NYHA functional class, their postoperative functional capacity was improved during the follow-up period (2.1±0.56 *vs*. 1.2±0.42; *P*=0.001).

## DISCUSSION

The main findings of the current study are that postoperative LVEF and NYHA functional class have significantly improved and postoperative LV end-systolic and end-diastolic diameters and left atrial diameter have significantly reduced during long-term follow-up.

Aortic root and valve replacement surgery has been applied since 1968 with reasonable low early/long-term follow-up, low mortality/morbidity rates, and improved long-term survival, in cases of annuloaortic ectasia, cystic medial necrosis, aortic aneurysm, and aortic dissection^[3]^. Aortic root replacement with composite graft is considered to be the choice of surgery for the diagnosis of ascending aorta aneurysm, annuloaortic ectasia, and aortic regurgitation^[3,14]^. The presence of Marfan syndrome, acute aortic dissection, endocarditis, redo surgery, urgent surgery, coronary artery disease, advanced age, low functional status, sudden cardiac death, congestive heart failure, stroke, chronic renal failure, and low cardiac output are considered to be indicators for early and late mortality rates^[15,16]^.

The reported mortality rate for elective Bentall surgery ranges from 1 to 5%^[17,18]^. In addition, Pacini et al.^[15]^ reported a 6.9% early-term mortality rate. Dhurandhar et al.^[1]^ reported an overall 5.9% early mortality rate (30 days) and a 3.6% elective surgery mortality. Furthermore, Pacini et al.^[15]^ reported a 274-patient study in that composite valve graft replacement was performed with a 6.9% early mortality rate. Mataraci et al.^[14]^ reported that the early mortality rate was 11.8% and the late mortality rate was 2.8%. We found similar results to those in our study, that early mortality rate was 8.3% and the late mortality rate was also 8.3%.

Hagl et al.^[19]^ reported mid/long-term results after Bentall procedure: overall survival rates in five years and eight years were 95% and 93%, respectively. Mataraci et al.^[14]^ reported long-term results of 254 patients: overall survival rates in one, three, and five years were 88.4%, 87.4%, and 84.5%, respectively, which was directly affected by the diagnosis of Marfan syndrome. Dhurandhar et al.^[1]^ reported that the overall survival rate was 84.4% in five years and 68.7% in 10 years. Pacini et al.^[15]^ reported five-year and 10-year survival rates as 77.7% and 63%, respectively. Patients with Marfan syndrome have a higher probability of redo surgery for residual aortic dilatation than those without Marfan syndrome (94.6% *vs*. 79.6% at 10-year follow-up, *P*=0.008)^[20]^. Our long-term survival results are comparable with previous studies: survival rates in five years and 10 years were 83.5% and 69.8%, respectively. Aortic dissection negatively affects the survival among patients who underwent aortic root replacement^[21]^, and for this reason aortic root replacement can be negligible during acute aortic dissection^[15,21]^.

Dhurandhar et al.^[1]^ reported that the most common etiology of patients who underwent Bentall procedure was congenital bicuspid aortic valves (28.3%), besides that, Marfan syndrome and annuloaortic ectasia constituted 19.3%.

Mataraci et al.^[14]^ reported that postoperative low cardiac output was revealed as the main etiology for in-hospital mortality (51.4%); in addition, they concluded that Marfan syndrome was the most common etiology, which affects the late mortality. However, there is no consensus on the influence of Marfan syndrome on long-term survival.

Patients with low ejection fraction may avoid aortic arch replacement. In the course of cooling and heating the patient during cardiopulmonary bypass, the total duration of the operation extends two hours, so the duration of myocardial ischemia increases. For this reason, there is a possibility of difficulty during weaning from the cardiopulmonary bypass, and length of İCU stay may increase.

Most of the studies investigated the surgical techniques and alterations, however, studies which investigate the LV function and condition on mid/long-term follow-up after the Bentall procedure are scarce. Dkojic et al.^[12]^ reported that long-term LV systolic function had been improved, and that LV mass was reduced after the Bentall procedure in patients who underwent elective surgery. Nardi et al.^[22]^ reported that LV function and LV end-diastolic diameter were significantly improved after the Bentall procedure in patients diagnosed with Marfan syndrome. Djokic et al.^[12]^ reported that LV mass was significantly reduced eight years after the Bentall procedure. Some authors advocated that Bentall procedure improves functional capacity in the long-term period^[23]^. LV systolic function was significantly increased and LV mass was significantly reduced after the Bentall procedure^[13]^.

Tanoue et al.^[13]^ investigated the LV performance after aortic valve replacement (AVR) in 263 patients with three parameters, such as contractility, efficiency, and ejection fraction. They found out that afterload and LV contractility were reduced in patients diagnosed with aortic valve stenosis, while they were increased in patients diagnosed with aortic valve regurgitation after AVR. However, LV efficiency was deteriorated in patients diagnosed with aortic regurgitation due to impaired LV function, albeit LV efficiency was ameliorated after AVR in patients diagnosed with both aortic valve stenosis and aortic valve stenosis-regurgitation. Furthermore, they demonstrated that the ejection fraction was significantly reduced after AVR in all patients^[13]^. In addition, LV volume reduced after AVR in patients with aortic valve regurgitation, which leads to ventricular contractility. Despite all these results, they concluded that LV contractility and efficiency were excellent and satisfactory after AVR during mid-term follow-up.

In another study, which was also conducted by Tanoue et al.^[24]^, 15 patients with annuloaortic ectasia with aortic regurgitation underwent Bentall surgery. They revealed that contractility and afterload were augmented and LV efficiency was significantly improved after Bentall procedure on mid-term follow-up.

Djokic et al.^[12]^ reported that patients with annuloaortic ectasia were more prone to be operated earlier than those without it. Sinus Valsalva influenced the hydrodynamic of native aortic valve leaflet motion^[24]^. Djokic et al.^[12]^ conducted a study with 90 patients who underwent elective Bentall procedure and investigated the LV systolic function, volume, and mass in the long-term period. They revealed that LV systolic function was increased while LV mass was reduced after the Bentall procedure on long-term follow-up (mean 117±41 months). Of those 90 patients, 66 patients were alive and four patients with acute myocardial infarction, four patients with heart failure, two patients with acute aortic dissection, and 13 patients with noncardiac causes passed away during follow-up.

Jiang et al.^[25]^ reported that the mean LV end-diastolic diameter reduced, while ejection fraction was increased after Bentall procedure in six patients. Our study shows similar results to those: postoperative LVEF was increased and LV end-systolic and end-diastolic diameters and left atrial diameter were significantly reduced after index surgery.

In addition, flanged Bentall technique is presumed to be an effective way to decrease postoperative bleeding from proximal anastomosis, and the flexibility and elasticity of the aortic annulus can be preserved, which reduces the tension. The advantage of mechanical composite valved graft is durability and it reduces reoperations due to aortic valve insufficiency^[25]^. The advantages of valve-sparing procedures, such as David reimplantation and Yacoub remodeling techniques, are avoiding anticoagulation therapy and reducing mechanical valve endocarditis and long-term risk of anticoagulation therapy^[26]^. However, no difference was found in terms of ventricular function and LV end-diastolic volume between these techniques. The mortality rate of patients with mechanical valve was found to be higher at the 10-year follow-up period, although there was no difference in mortality in their 5-year follow-up^[26,29]^.

In our experience and surgical notion, we believe that flanged procedure provides lower anastomatic hemorrhage and pseudoaneurysm than button procedure, and that internal organs are better preserved at 18ºC. We did not dare to cool down below 18ºC due to further elongation of surgery to prevent and reduce the destruction of blood cells as much as possible.

This study has a number of limitations worth noting. Firstly, we conducted a retrospective study, including patients who underwent flanged or button Bentall procedure and some factors may have changed during long-term follow-up. Secondly, the number of patients included in our study may be relatively small compared to other studies. Thirdly, it’s a single-center design. Fourthly, only diameters and ejection fraction values were available, except ventricular volumes and LV mass, in this retrospective study. And finally, the effect of drugs was not examined on LV function.

## CONCLUSION

The Bentall procedure significantly improved LV systolic function and condition and decreased LV end-systolic and end-diastolic diameters and left atrial diameter on long-term follow-up, based on the transthoracic echocardiography. The Bentall procedure can be performed with reasonable mortality and morbidity rates on long-term follow-up.

**Table:** 

Authors’ roles & responsibilities	
SBD	Writing, design, analysis; final approval of the version to be published
MKD	Substantial contributions to the conception or design of the work; final approval of the version to be published
SMY	Interpretation of data for the work; final approval of the version to be published
UY	The acquisition; final approval of the version to be published
MMG	Substantial contributions to the conception or design of the work; final approval of the version to be published
MP	Substantial contributions to the conception or design of the work; final approval of the version to be published
FK	Writing; final approval of the version to be published
HTK	Analysis; final approval of the version to be published
